# Screening copy number variations in 35 unsolved inherited retinal disease families

**DOI:** 10.1007/s00439-023-02631-4

**Published:** 2024-01-29

**Authors:** Xiaozhen Liu, Hehua Dai, Genlin Li, Ruixuan Jia, Xiang Meng, Shicheng Yu, Liping Yang, Jing Hong

**Affiliations:** 1 Department of Ophthalmology, Peking University Third Hospital, Beijing, 100191 China; 2 Beijing Key Laboratory of Restoration of Damaged Ocular Nerve, Peking University Third Hospital, Beijing, 100191 China; 3 Department of Ophthalmology, The First Affiliated Hospital of Shandong First Medical University & Shandong Provincial Qianfoshan Hospital, Jinan, 250014 China; 4 Beijing Tongren Eye Center, Beijing Tongren Hospital, Capital Medical University, Beijing Ophthalmology & Visual Sciences Key Lab, Beijing, 100730 China

## Abstract

The purpose of this study was to screen Copy Number Variations (CNVs) in 35 unsolved Inherited Retinal Dystrophy (IRD) families. Initially, next generation sequencing, including a specific Hereditary Eye Disease Enrichment Panel or Whole exome sequencing, was employed to screen (likely) pathogenic Single-nucleotide Variants (SNVs) and small Insertions and Deletions (indels) for these cases. All available SNVs and indels were further validated and co-segregation analyses were performed in available family members by Sanger sequencing. If not, after excluding deep intronic variants, Multiplex ligation-dependent probe amplification (MLPA), quantitative fluorescence PCR (QF-PCR) and Sanger sequencing were employed to screen CNVs. We determined that 18 probands who had heterozygous SNVs/indels or whose parents were not consanguineous but had homozygous SNVs/indels in autosomal recessive IRDs genes had CNVs in another allele of these genes, 11 families had disease-causing hemizygous CNVs in X-linked IRD genes, 6 families had (likely) pathogenic heterozygous CNVs in *PRPF31* gene. Of 35 families, 33 different CNVs in 16 IRD-associated genes were detected, with *PRPF31*, *EYS* and *USH2A* the most common disease-causing gene in CNVs. Twenty-six and 7 of them were deletion and duplication CNVs, respectively. Among them, 14 CNVs were first reported in this study. Our research indicates that CNVs contribute a lot to IRDs, and screening of CNVs substantially increases the diagnostic rate of IRD. Our results emphasize that MLPA and QF-PCR are ideal methods to validate CNVs, and the novel CNVs reported herein expand the mutational spectrums of IRDs.

**Supplementary Information:**

The online version contains supplementary material available at 10.1007/s00439-023-02631-4.

## Introduction

Inherited retinal dystrophies (IRDs) are a group of monogenic diseases characterized by the progressive degeneration of photoreceptors or the retinal pigment epithelial (RPE) cells (Berger et al. 2010; Liu et al. 2021), which consist of over 20 different clinical phenotypes (Berger et al. 2010) involving more than 300 genes (RetNet; https://sph.uth.edu/retnet/).

Next-generation sequencing (NGS), including targeted region capture sequencing and whole exome sequencing (WES), is widely and efficiently used to confirm the genetic causes of IRDs (Lee and Garg 2015; Liu et al. 2021; Stone et al. 2017). However, despite substantial progress in NGS technology and the discovery of new IRD genes, disease-causing mutations are merely detected in approximately 50–76% of IRD cases (Consugar et al. 2015; Lee and Garg 2015; Liu et al. 2021; Stone et al. 2017). An important reason is that most previous studies on IRDs are prone to focus on single-base substitution mutations or small insertions and deletions (indels). There are several factors which may influence the genetic diagnostic rate as well. Aside from the fact that a new disease-causing gene might be involved, some deep-intronic sequence changes, mutations in regulatory regions, and structural variations (SVs) such as internal inversions (or chromosomal rearrangements) with loss or gain of genes/exons, copy number variations (CNVs) might also be missed (Liu et al. 2021). CNV is referred to as large insertions and deletions (Liu et al. 2012), which is recognized as an important contributor to the genetic causality of IRDs (Bujakowska et al. 2017). A previous study reported that CNV contributed to 9% of pathogenicity in IRD cases. Our study also indicated that CNV contributed to 3.00% of total panel-based positive diagnosis cases (Liu et al. 2021). Although CNV has become increasingly recognized as a potential key genetic cause of IRDs, the methods of screening for CNVs, such as multiplex ligation-dependent probe amplification (MLPA) and quantitative fluorescence PCR (QF-PCR), etc*.* are not used as a first-tier test for IRD patients.

In this study, we aimed to investigate potential CNVs in 35 IRD unsolved cases. In the first tier, we used NGS, including WES or a specific hereditary eye disease enrichment panel (HEDEP) to screen the disease-causing Single-nucleotide Variants (SNVs) and small indels for these families. However, no disease-causing genetic causes in these cases were confirmed. Further, for patients whose high throughput sequencing data indicated CNVs, or patients who had a heterozygous variant in autosomal recessive (AR) IRD genes, or whose parents were not consanguineous but had a homozygous mutation in AR genes, CNVs were validated by MLPA or QF-PCR. Additionally, patients affected with red-green Color Vision Deficiency (CVD) were submitted to Sanger sequencing to analyze CNVs in the *OPN1LW* and *OPN1MW* gene. In total, 33 different CNVs in 16 different IRD genes were confirmed in these 35 families, with *PRPF31*, *EYS*, and *USH2A* being the most common gene with CNVs. Among them, 14 novel CNVs were reported. Our study indicated that CNV was an important contributor to IRDs and emphasized the potential role of CNV screening as diagnostics for the diseases.

## Materials and methods

### Ethics statement

This study conformed to the tenets of the Declaration of Helsinki. All experiments involving patients' DNA and the DNA of their relatives were approved by the Peking University Third Hospital Medical Ethics Committee (No. 2012093). Written informed consent was obtained from all participants or from guardians on behalf of minors. The ethics committee approved this consent procedure.

### Patients

This study recruited 35 unrelated Chinese families of Han ethnicity affected with IRDs, including Retinitis Pigmentosa (RP) and Bietti crystalline corneoretinal dystrophy (BCD), Cone rod dystrophy (CORD), etc. from the Department of Ophthalmology, Peking University Third Hospital and Beijing Tongren Hospital.

Detailed medical and family histories were obtained from probands or their family members. All patients underwent standard ophthalmic examinations, including best corrected visual acuity (BCVA), slit-lamp biomicroscopy, intraocular pressure measurement, dilated indirect ophthalmoscopy and fundus photography, electroretinography (ERG), and visual field tests, if possible. The patients underwent a systematic physical examination before genetic testing. Inheritance patterns were classified based on criteria described by Stone et al. with minimal modification (Stone et al. 2017): (1) autosomal dominant (AD; a minimum of three generations with at least one instance of male-to-male transmission); (2) AR (several affected patients in a single sibship with healthy parents); (3) X-linked (affected males in multiple sibships connected through unaffected or mildly affected females and no male-to-male transmission); (4) uncertain inheritance (sporadic patients and other multiplex kindreds).

### Molecular genetics analysis

Blood samples were obtained from all probands and their available family members. Genomic DNA (gDNA) was extracted using standard protocols ((D2492, Omega Bio-Tek). The probands of 35 families were subjected to HEDEP for targeted exon enrichment analysis, which could capture 483 IRD genes, or the WES, which was done using Agilent’s SureSelect Human All Exon V6 Kit (Agilent, USA) (Liu et al. 2021). NGS was performed using an Illumina HiSeq X platform (Illumina, San Diego, California). HEDEP and WES sequencing data were analyzed as previously described (Liu et al. 2021; Zhang et al. 2016).

### High throughput data analysis and variant classification

HEDEP sequencing data were analyzed as described previously (Liu et al. 2021). Sequence changes were classified according to the American College of Medical Genetics and Genomics (ACMG) and the Association for Molecular Pathology (AMP) variant interpretation guidelines (Richards et al. 2015). In this study, only variants identified as pathogenic or likely pathogenic ((likely) pathogenic) were reported. Those of uncertain significance, likely benign, or benign were not included.

### Sanger sequencing

For patients in whom only one (likely) pathogenic variant was detected in a gene responsible for a recessive IRD, and if the intronic variants in this recessive gene were described previously, Sanger sequencing of the specific intronic regions of the *ABCA4* (IVS5 c.570 + 1798A > G; IVS13 c.1938-619A > G; IVS14 c.2160 + 584A > G; IVS20 c.3050 + 370C > T; IVS30 c.4539 + 1729G > T & c.4539 + 2064C > T; IVS36 c.5196 + 899C > T & c.5196 + 1015A > G; and IVS44 c.6148-471C > T) and *USH2A* (IVS27 c.5573-834A > G; IVS40 c.7595-2144A > G; IVS44 c.8845 + 628C > T; and IVS50 c.9959-4159A > G) genes was also performed. For five patients who were affected with CVD or had a CVD family history, Sanger sequencing was performed to detect the disease-causing variants in the *OPN1LW* and *OPN1MW* genes, primers are listed in Table S1.

All variants considered (likely) pathogenic in this study were validated by Sanger sequencing. Sanger sequencing was also used to determine whether the variant was co-segregated with the disease phenotype in available family members.

### MLPA or QF-PCR

For patients whose high throughput sequencing data indicated CNVs, CNVs were validated by MLPA or QF-PCR as described in our previous study (Liu et al. 2021). Briefly, *CYP4V2* MRC-Holland kit P149-025R, *ABCA4* MRC-Holland kit P151-025R and P152-025R, *LCA* MRC-Holland kit P221-025R and P222-025R, Retinitis MRC-Holland kit P235-025R, *PCDH15* MRC-Holland kit P292-025R, *EYS* MRC-Holland kit P328-25R, *USH2A* MRC-Holland kit P361-025R and P362-025R, *CHM*-RP2-*RPGR* P366-025R, and *BEST1*-*PRPH2* MRC-Holland kit P367-025R MLPA kits (MRC-Holland, Amsterdam, Netherlands) were used to detect CNVs, as suggested by the manufacturer. Amplification products were run on an ABI 3730xl sequencer (Carlsbad, CA, USA), and the results were analyzed using software supported by *Coffalyser.Net*. Primers for QF-PCR were designed to amplify each exon of the disease-causing inherited eye disease genes (Table S1); the product length was ⁓200 bp. The *ALB* gene was used as the internal reference. Each 20 μl reaction contains 10 μl of TransStart Top Green qPCR SuperMix (TransGen Biotech), 0.25 μM of each forward and reverse primer, and 50 ng blood DNA with each sample run in duplicate. The qPCR reactions were carried out by the ABI7500 Real-Time PCR Detection System (Carlsbad, CA, USA). Relative quantification of each amplicon in the proband, their available parents or relatives and an unrelated control were calculated by comparing it to the internal reference gene. By comparing the fold-change differences between the control and the proband’s samples (ΔΔCt), the relative level of each amplicon was determined (2^−ΔΔCt^). The standard deviation reflecting normalization to the reference gene was calculated and presented as the error bars.

## Results

### The flow path of CNV detection

**Fig. 1:**
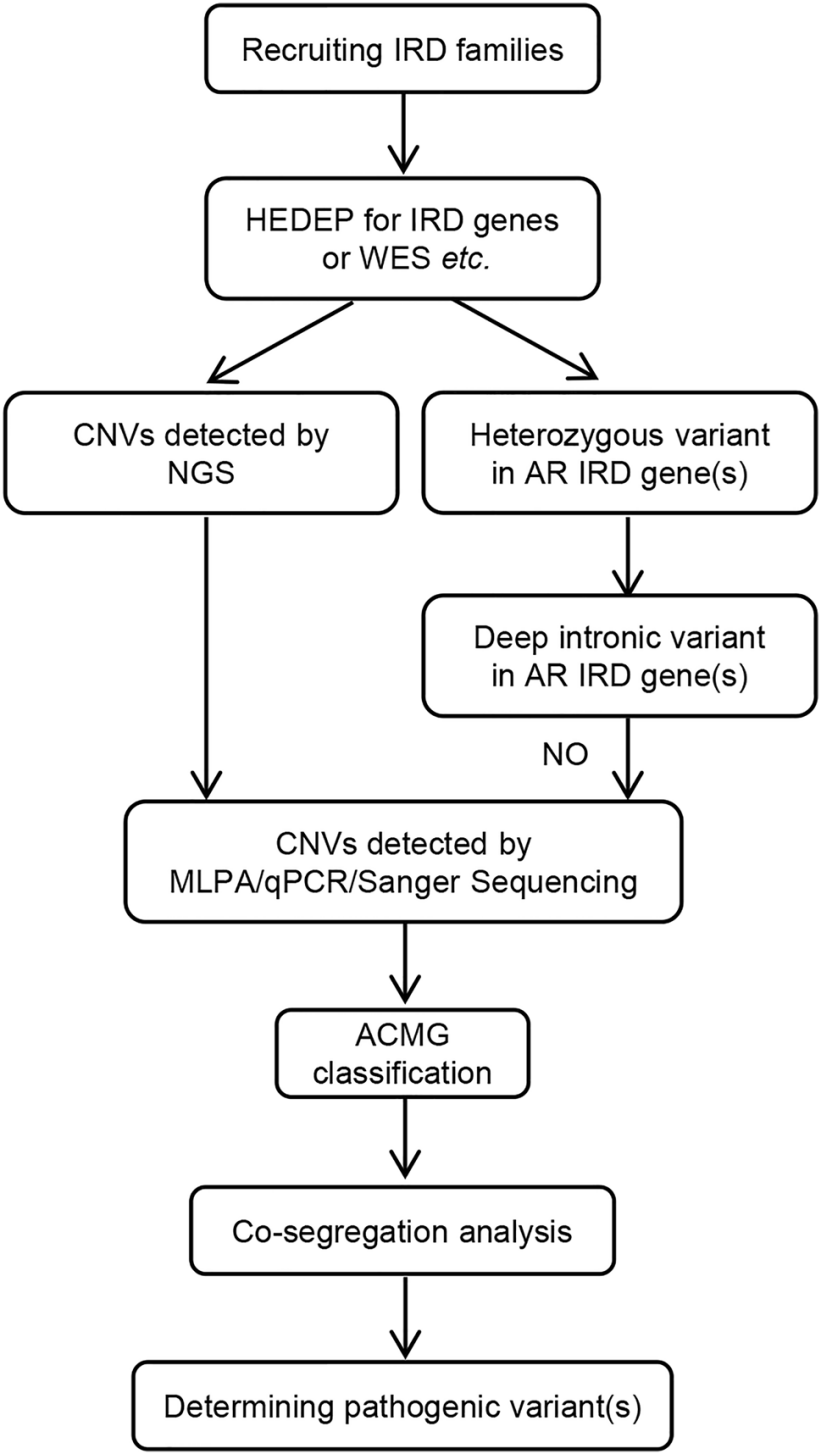
Flowchart of screening CNVs in this study. *AR* autosomal recessive, *CNV* copy number variation, *HEDEP* hereditary eye disease enrichment panel, *IRD* inherited retinal dystrophy, *NGS* next-generation sequencing

Thirty-five probands affected with IRDs were sequenced by NGS technology (WES or HEDEP) and analyzed with comprehensive genetic testing to detect SNVs and indels, as described previously (Liu et al. 2021). However, these cases were unsolved. Among them, 14 cases were found to have a heterozygous SNV/indel in AR IRD genes, 4 probands in unconsanguineous families to have pathogenic homozygous SNV/indel in AR IRD genes while the proband acquired the same mutation from her parents with a slight chance, and no pathogenic SNV/indels in 16 families were detected. Besides, although compound heterozygous variants (p.Gly1961Arg and p.Gly469Arg) in *ABCA4 *in Family P30 were detected, variant p.Gly469Arg was of uncertain significance (US). As illustrated in Fig. 1, (a) if high throughput sequencing data of HEDEP indicated CNVs (Fig. S1), MLPA or QF-PCR would be employed to validate CNVs; (b) if unsolved families had a heterozygous SNV or indel in AR IRD genes, deep intronic variant(s) in these genes would be analyzed, if not, CNVs would be studied further; (c) if patients in unconsanguineous families had homozygous SNV/indel in IRD genes, CNVs would be studied further as well; (d) especially, CNVs in *OPN1LW* and *OPN1MW* genes in five families affected with CVD were also analyzed using Sanger sequencing.

### The results of the CNV analysis

**Fig. 2:**
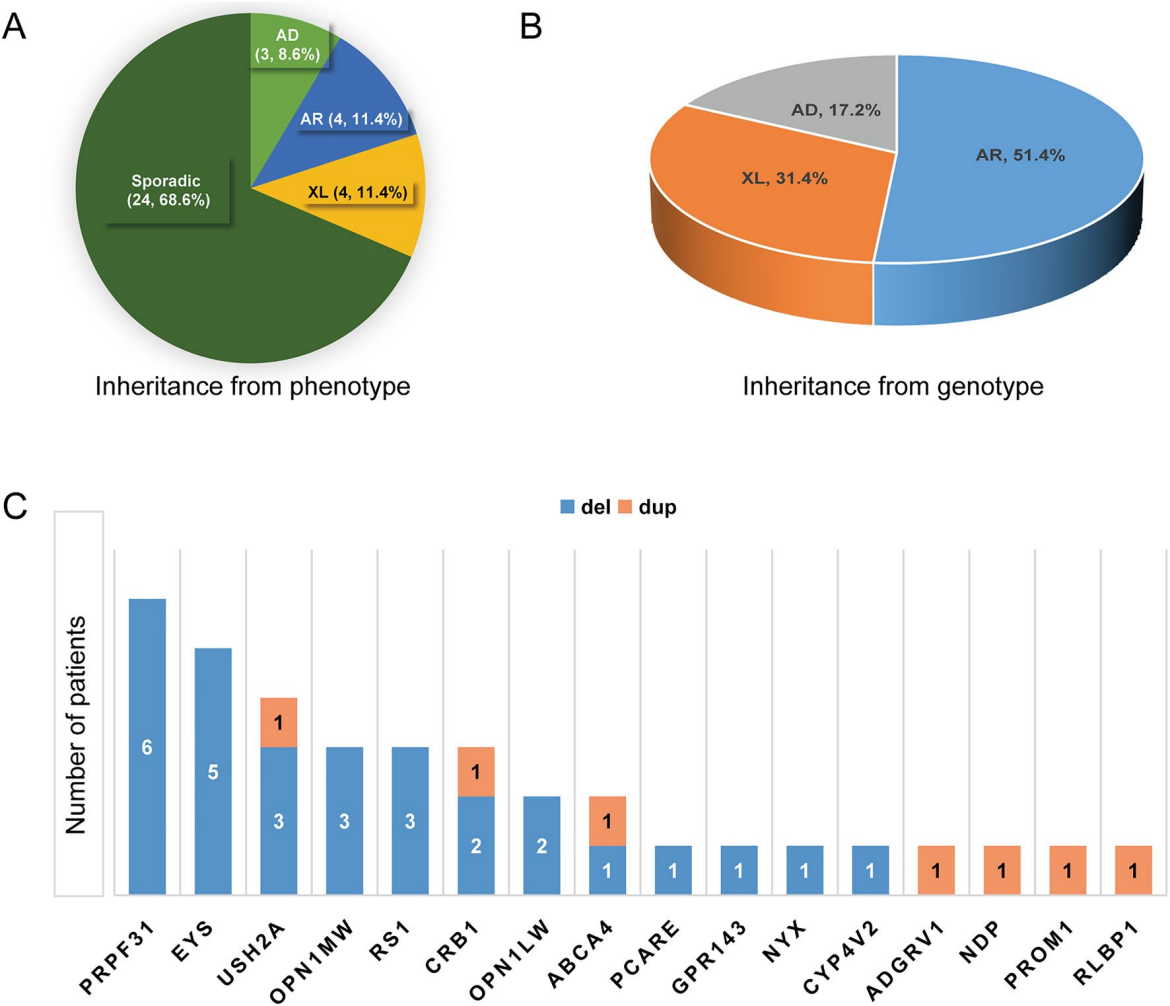
The clinical information and (likely) pathogenic CNVs in 35 IRDs families. **a** The percentage of different inheritance modes from clinical information; **b** The percentage of different inheritance modes from patients’ genotypes; **c** (Likely) pathogenic CNVs in 16 IRD-associated genes of 35 families

**Fig. 3:**
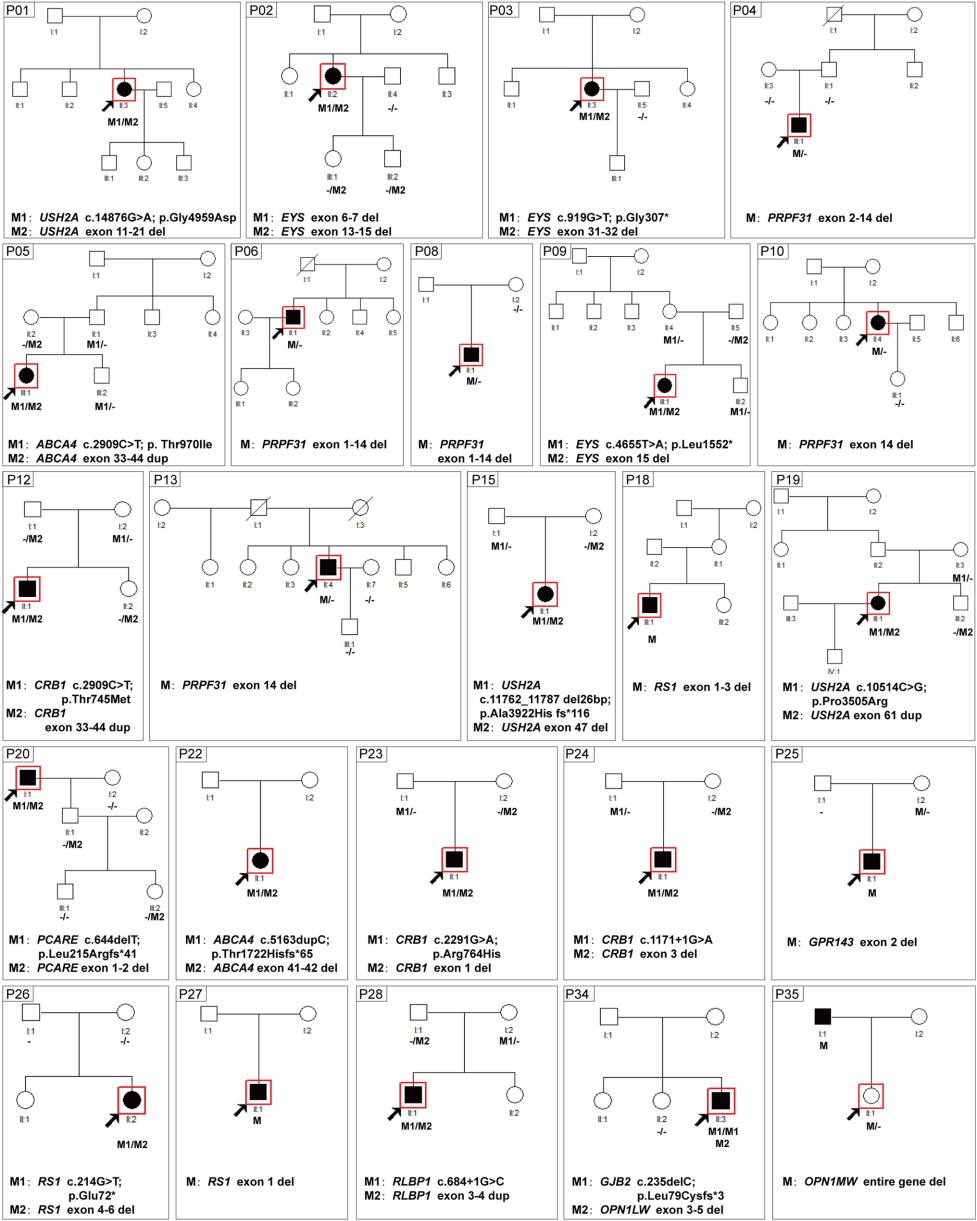
Pedigrees, genotypes, and segregation analysis for all sporadic IRDs families. Affected individuals are given with filled symbols; unaffected individuals are represented with *open symbols* and individuals with a milder phenotype are given with gray symbols. *Arrows* indicate the proband. DNA numbers and genotypes for individuals with available DNA are given below each symbol

**Fig. 4:**
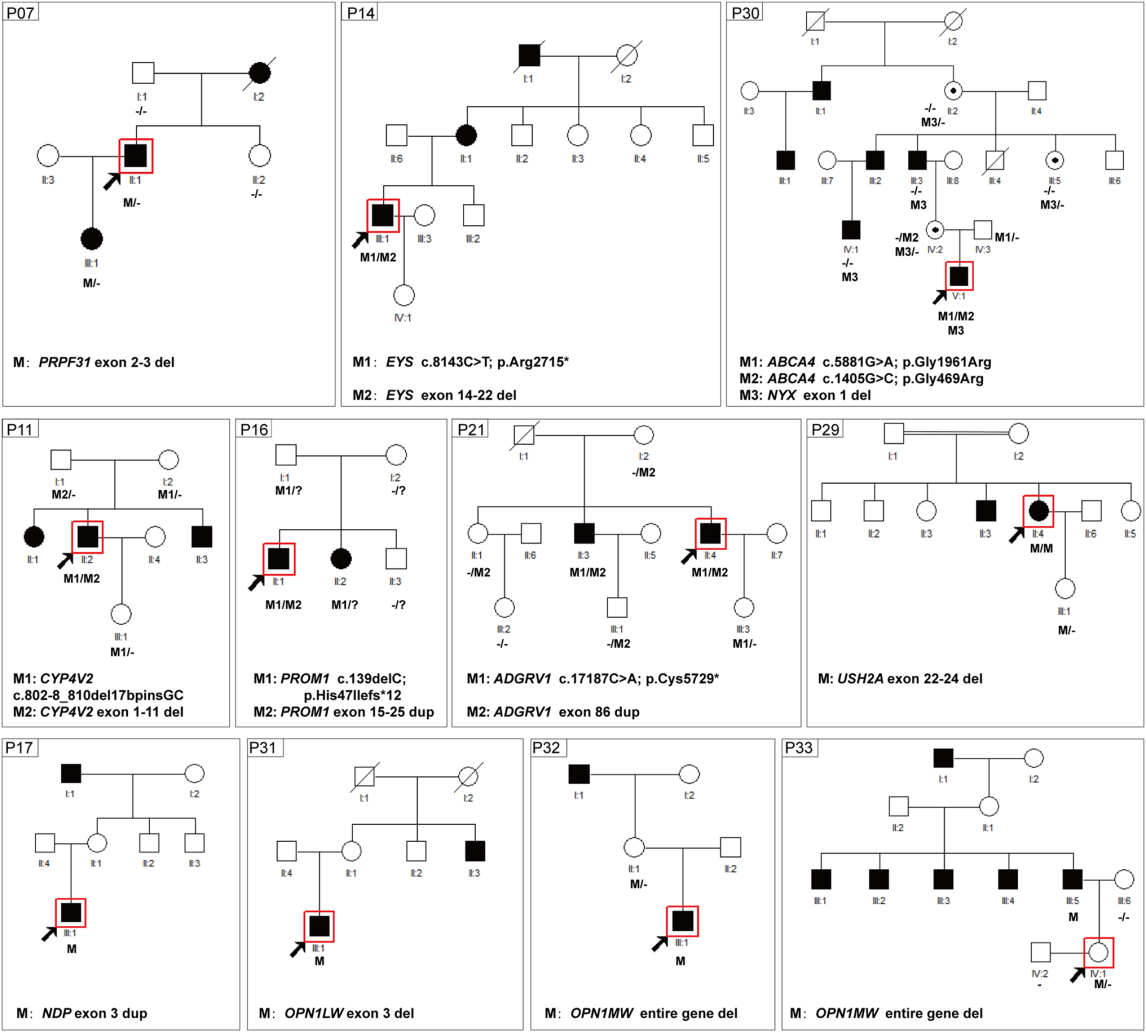
Pedigrees, genotypes, and segregation analysis for AR, AD, XL IRDs families. Affected individuals are given with filled symbols; unaffected individuals are represented with *open symbols* and individuals with a milder phenotype are given with gray symbols. *Arrows* indicate the proband. DNA numbers and genotypes for individuals with available DNA are given below each symbol. *AR* autosomal recessive, *AD* autosomal dominant, *XL* X-linked

According to the initial clinical diagnosis, patients had nine different IRD phenotypes, with RP as the most common form (Table S2). In most cases (24/35, 68.6%), a family had only one affected individual (sporadic cases, Figs. 2a and 3). Whereas based on family pedigree, in 8.6% (3/35) an AD, in 11.4% (4/35) an AR and in 11.4% (4/35) an X-linked inheritance was predicted (Figs. 2a and 4). The genetic testing results indicated that 18 families (51.4%), 11 families (31.4%) and six families (17.2%) had pathogenic variants in AR, XL and AD IRD-related genes, respectively (Fig. 2b). In 24 sporadic cases, the genetic testing indicated that 13 cases had disease-causing mutations in AR IRD gene, 5 cases in AD IRD genes, 6 cases in XL IRD genes, thereby, their inheritance mode were redefined. Importantly, the proband of Family P34 had deafness and CVD, HEDEP indicated that he had a homozygous deletion variant (p. Leu79Cysfs*3) in *GJB2* gene, which has been reported to cause AR deafness(Lin et al. 2022; Xia et al. 2019), while no CNV in *GJB2* gene was detected by QF-PCR. The family denied consanguineous marriage, and his elder sister did not have this mutation, we wonder whether the mutation occurred only in the proband but was inherited from his parents. Sanger sequencing indicated his CVD was caused by a hemizygous CNV (exon 3–5 del) in *OPN1LW.* In 3 AD cases (Fig. 4, P07, P14, P30), Family P07 had a heterozygous CNV (exon 2–3 del) in *PRPF31*, the inheritance mode was AD. Family P14 had compound heterozygous variants (p. Arg2715* and exon 14–22 del) in *EYS*, and the inheritance mode was redefined as AR. The proband of Family P30 was a 2-year-old boy, the current clinical information provided by his mother showed that he had high myopia (-7D) bilaterally, the boy had compound heterozygous variants (p. Gly1961Arg and p. Gly469Arg) in *ABCA4* which were inherited from his father and mother, respectively, and a hemizygous CNV—exon1 del in *NXY* inherited from mother. According to ACMG guidelines, p. Gly469Arg was of US. However, the boy was too little to confirm whether he would have *ABCA4*-associated phenotypes, so it was ideal to follow-up on his disease progression. Considering the uncertain pathogenicity of p. Gly469Arg, it was not appropriate to define their AR inheritance mode, thereby, the inheritance pattern should be XL. The genetic testing results indicated that four AR families (Fig. 4, P11, P16, P21, P29) had pathogenic variants in AR IRD genes, their inheritance mode was AR. Among them, a consanguineous family had a homozygous CNV (exon 22–24 del) in *USH2A*. In four XL families (Fig. 4, P17, P31, P32, P33), hemizygous CNVs in three XL IRD genes were detected, and their pattern modes were XL.

As illustrated in Fig. 2c, 35 families had 36 CNVs; 2 cases had the same CNV (exon 14 del) in *PRPF31*, and 3 cases had the same CNV (entire gene del) in *OPN1MW*. Thereby, 33 different CNVs in 16 IRD-associated genes were found (Table S2 and S3), 26 and 7 of them were deletions and duplication CNVs, respectively. Herein, *PRPF31* (five different CNVs in six cases), *EYS* (five different CNVs in four cases), *USH2A* (four different CNVs in four cases) were the most common disease-causing genes in CNVs (Fig. 2c), and CNVs in them were all deletion variants. Among them, 14 CNVs were first reported in this study.

### CNVs analysis by MLPA

Sixteen unsolved probands and their 53 family numbers were subjected to MLPA to screen CNVs, 13 deletion CNVs and 3 duplication CNVs were found (Figs. 3 and 4, Table S2).

**Fig. 5:**
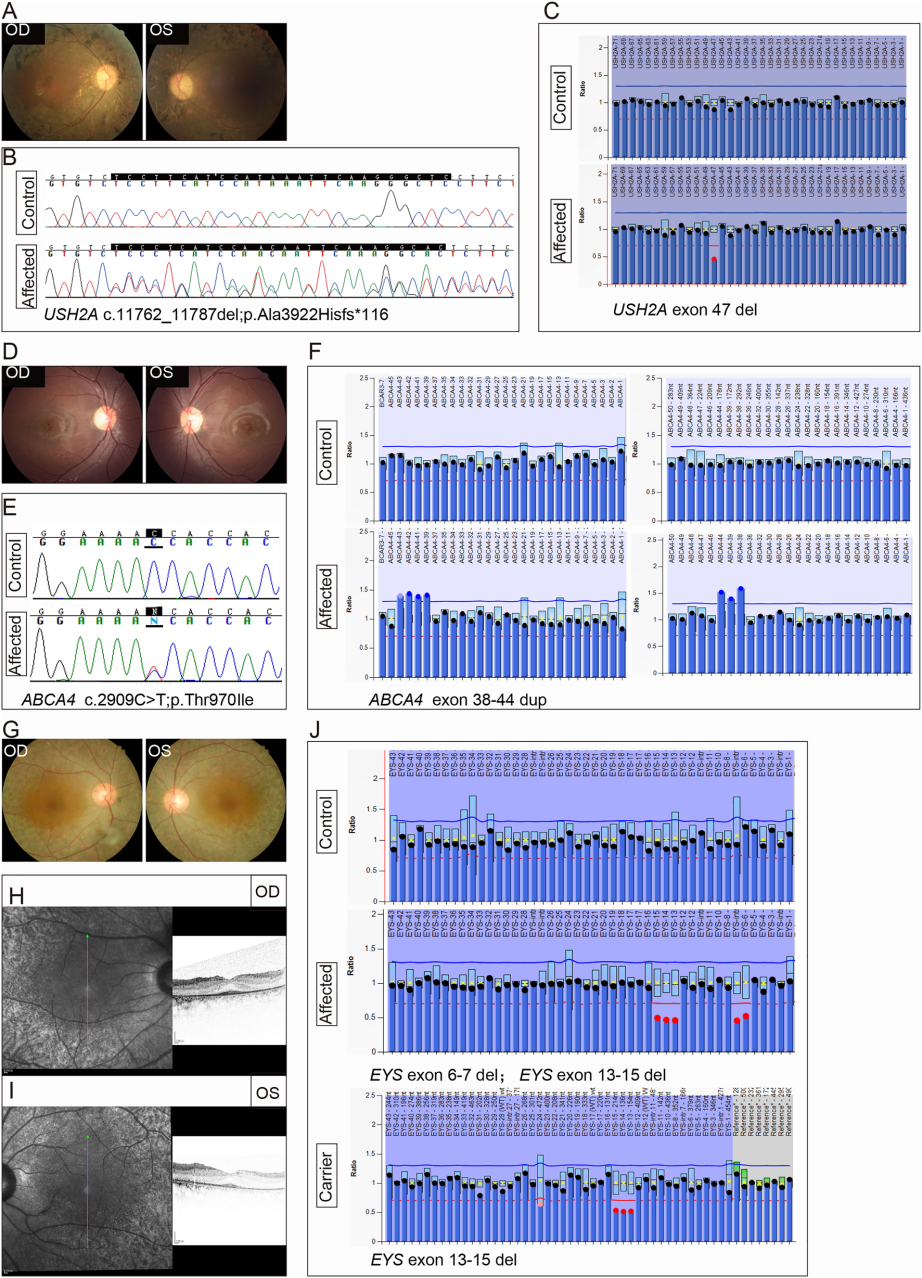
Identification of CNVs of Family P15, P05 and P02. Fundus photograph of both eyes (**a**), a frameshift mutation (c.11762_c.11787 del; p. Ala3922His fs*116, **b**) by Sanger sequencing and a CNV (exon 47 del, **c**) by MLPA in *USH2A* gene were determined in proband of Family P15. Fundus photograph of both eyes (**d**), a missense mutation (c.2909C > T; p. Thr970Ile, **e**) by Sanger sequencing and a CNV (exon 38–44 dup, **f**) by MLPA in *ABCA4* gene were determined in proband of Family P05. Fundus photograph of both eyes (**g**), OCT image of both eyes (**h** and **i**), compound heterozygous CNVs (exon 6–7 del and exon 13–15 del, **j**) by MLPA in *EYS* gene were determined in proband of Family P02

The proband of Family P15 was an 18-year-old girl, she was affected with night blindness and hearing impairment when she was 4, her visual acuity and hearing became worse with age, her BCVA was 0.4 bilaterally, and she had tunnel vision when she was subjected to HEDEP. Her fundus photograph revealed typical RP phenotypes, including bone spicule deposits, attenuated retinal blood vessels, and optic disc pallor (Fig. 5a) (Zhang et al. 2016). Sanger sequencing indicated that she had a heterozygous variant c.11762_11787 del26bp (p.Ala3922His fs*116) in *USH2A*, inherited from his father (Figs. 3, P15 and 5b). MLPA result indicated that she harboured a CNV—exon 47 del in *USH2A*, inherited from her mother (Figs. 3, P15 and 5c).

The proband of Family P05 was affected with STGD, she was an 11-year-old girl who complained of visual acuity damage from her childhood. Her BCVA was 0.8 in both eyes. Fundus examination revealed bilateral beaten-bronze-appearing macular lesions with yellow flecks around the macula (Fig. 5d). Sanger sequencing indicated that she had a heterozygous mutation—c.2909C > T (p. Thr970Ile) in *ABCA4*, inherited from her father (Figs. 3, P05 and 5e). MLPA result showed that she also had a disease-causing CNV—exon 38–44 dup in *ABCA4* (Figs 3, P05 and 5f).

The proband of Family P02 was affected with CORD, she was a 42-year-old woman who complained of visual acuity damage from her 10 s. Her BCVA was 0.3 in both eyes. Fundus examination revealed an irregular RPE atrophy centred on fixation surrounded by a circular atrophied area 4 mm in diameter in both eyes (Fig. 5g). The optical coherence tomography (OCT) scan showed a slight reduction of foveal thickness and a bilateral disruption of the photoreceptor layer (Fig. 5h–i). HEDEP results detected no disease-causing SNVs or indels in this proband. MLPA result indicated she had two CNVs, including exon 6–7 del and exon 13–15 del in *EYS* (Fig. 5j). To confirm whether these two CNVs were in an allele, her husband, son, and daughter were subjected to MLPA. The results demonstrated that her husband was unaffected, the two children had the heterozygous CNV, exon 13–15 del in *ABCA4* (Figs. 3, P02 and 5j, carrier), implying that the two CNVs in the proband were in two alleles, therefore, the two heterozygous CNVs led to her AR CORD phenotypes.

**Fig. 6:**
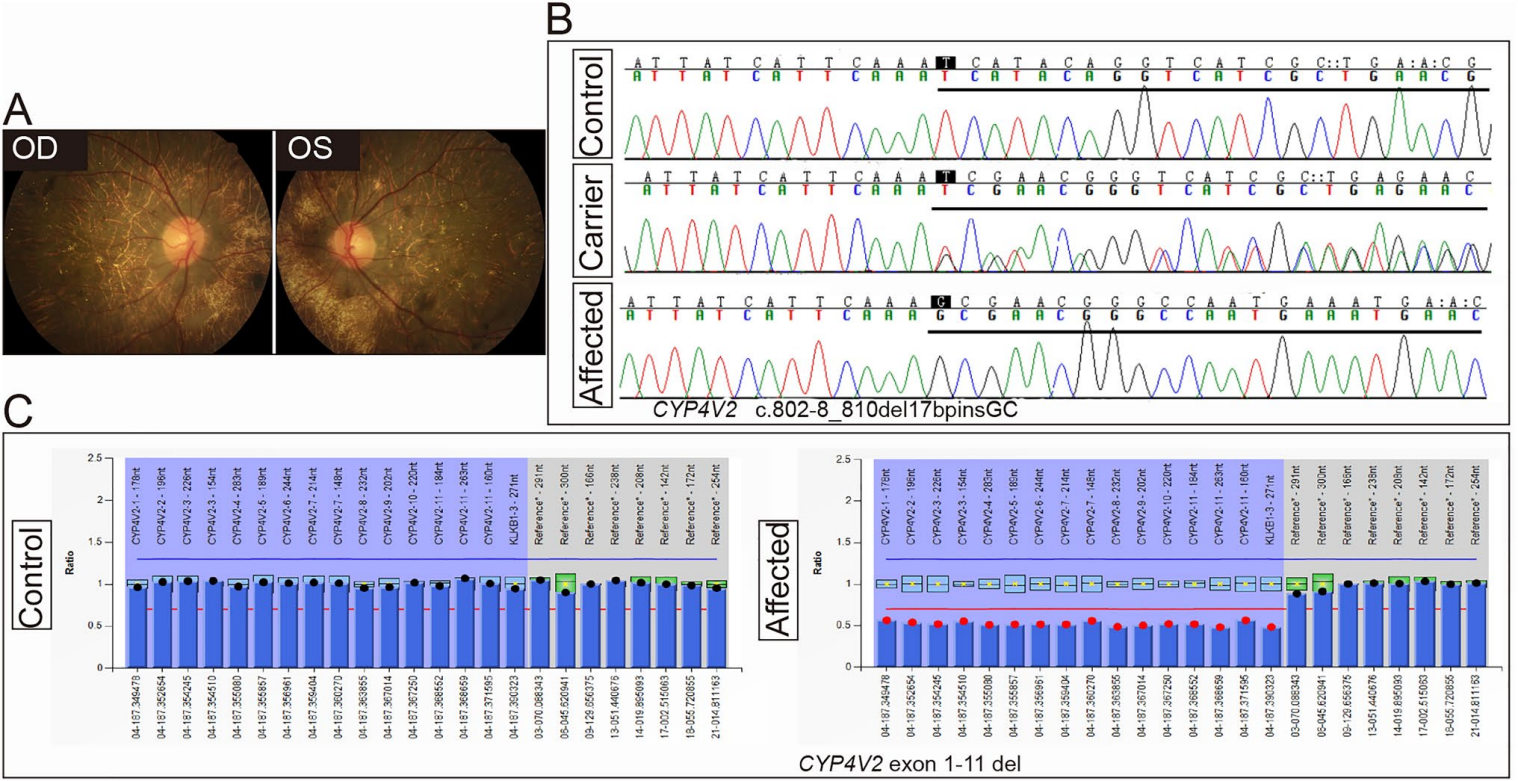
Identification of CNV of Family P11. Fundus photograph of both eyes (**a**), an intronic variant (c.802-8_810del17bpinsGC, **b**) by Sanger sequencing and a CNV (exon 1–11 del, **c**) by MLPA in *CYP4V2* gene were determined in the proband

According to clinical information, the inheritance type of Family P11 (Fig. 4, P11) was AR, and the proband was diagnosed with BCD. The proband, a 41-year-old man, complained of poor visual acuity since he was 10 and suffered from progressive vision loss, his BCVA was 0.1 and 0.02 in the right and left eye, respectively. Fundus examination showed small yellow-white glittery crystalline deposits located throughout the posterior pole bilaterally (Fig. 6a). Sanger sequencing indicated the proband had a homozygous variant c.802-8_810del17bpinsGC in *CYP4V2*, which was inherited from her mother and was passed to her unaffected daughter (Figs. 4, P11 and 6b). While family P11 was not consanguineous, the proband acquired the same mutation from her parents with a slight chance, suggesting that we should further identify the reason of homozygosity of her pathogenic mutation. Then, no deep-intronic variants was not found in her, MLPA was employed to screen CNV in *CYP4V2*, the result indicated the proband carried a heterozygous CNV—exon 1–11 del in *CYP4V2*, which was inherited from her father (Figs. 4, P11 and 6c), demonstrating that the large-fragment gene deletion in an allele would make the variant in another allele homozygous. Taken together, HEDEP in combination of MLPA and co-segregation analysis in Family P11 help us to confirm that the compound heterozygous variants in the proband resulted in her AR BCD phenotypes.

## CNVs analysis by QF-PCR

**Fig. 7:**
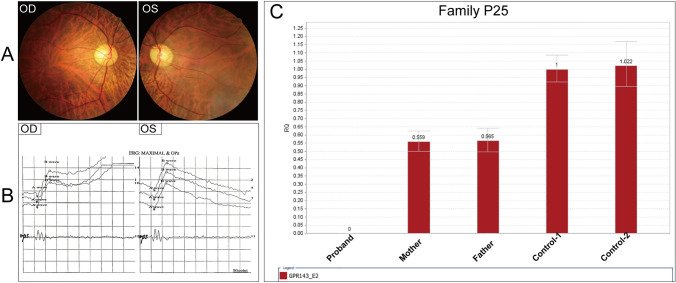
Identification of CNV of Family P25. Fundus photograph of both eyes (**a**), electroretinogram result in both eyes (**b**), and a hemizygous CNV (exon 2 del, **c**) in *GPR143* gene were determined by QF-PCR in the proband

Fourteen probands and their 53 family numbers were subjected to QF-PCR to screen CNVs (Figs. 3 and 4). Ten different deletion CNVs and four duplication CNVs were found. The proband of Family P25 was a 31-year-old man, he suffered from poor vision acuity, refractive error, amblyopia, and nystagmus when he was one, and his BCVA was 0.25 bilaterally presently. His fundus photograph revealed RP phenotypes, including bone spicule deposits and optic disc pallor (Fig. 7a). His ERG result showed mildly reduced a- and b-waves (Fig. 7b), and the ERG result was disturbed by his nystagmus phenotype. Initially, he was diagnosed with nystagmus and RP. His HEDEP result showed that he had a CNV—exon 2 del in the *GPR143* gene, which was inherited from his mother (Fig. 2 P25, Fig. S1B, Table S2). The QF-PCR result confirmed the NGS result (Fig. 7c), showing that the proband had the hemizygous CNV and his mother had the heterozygous CNV, while two female healthy controls were unaffected. The *GPR143* gene consists of 9 exons spanning approximately 40 kb on chromosome Xp22.3 (Han et al. 2015). To date, over 100 mutations (Human Gene Mutations Database, http://www.hgmd.cf.ac.uk) in this gene have been reported to cause ocular albinism type I (OA1) (Bassi et al. 1995), and previous studies have been reported that some mutations in *GPR143* cause X-linked congenital nystagmus in Chinese families (Han et al. 2015; Hu et al. 2011; Liu et al. 2007; Peng et al. 2009). Herein, the proband did not complain of night blindness and did not present with OA1 phenotypes. Thereby, his clinical diagnosis was redefined as X-linked congenital nystagmus.

### CNVs analysis by Sanger sequencing

**Fig. 8:**
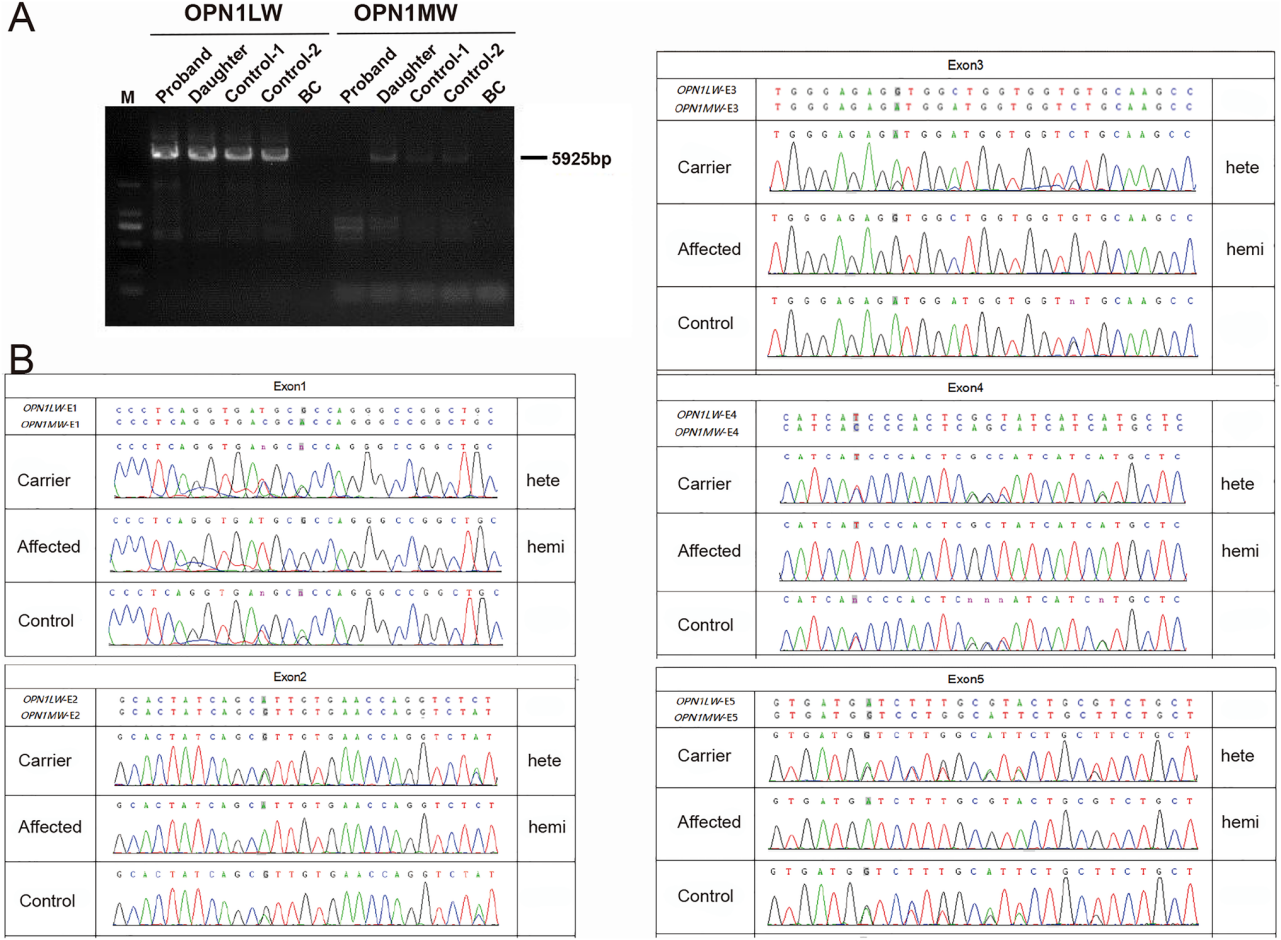
Identification of CNV in *OPN1LW/OPN1MW* gene of Family P33. **a** Gel electrophoresis results of the proband, his unaffected daughter, and two male control samples for the entire *OPN1LW/OPN1MW* gene. **b** Sanger sequencing results of the five exons of *OPN1LW/OPN1MW* gene. The results indicated the affected proband carried a hemizygous CNV (the entire gene del) in *OPN1MW* gene, his daughter (carrier) had the heterozygous CNV

Five probands with a history of CVD and their 11 family numbers were subjected to Sanger sequencing to screen CNVs (Figs. 3 and 4, Table S2). Three deletion CNVs in the *OPN1LW* and *OPN1MW* genes in these five cases were found. It is worth mentioning that in Family P35 (individual II:1) and P33 (individual IV:1), two unaffected females were the first in their family to ask for genetic testing because their father or brothers were affected with CVD (Figs. 3, P35 and 4, P33). According to clinical information, the inheritance pattern of Family P31, P32, and P33 was X-Linked, while the inheritance pattern of Family P34 and P35 was sporadic. The genetic testing indicated that three of them had the entire gene deletion of the *OPN1MW* gene (Figs. 3 and 4, and Table S2), this CNV has been reported to cause CVD (Ayyagari et al. 2000; Deeb et al. 1992). The PCR gel electrophoresis result (Fig. 8a) and Sanger sequencing (Fig. 8b) indicated that in Family P33, the proband (individual III:5) had the hemizygous CNV—entire gene deletion of the *OPN1MW* gene, his daughter (individual IV:1) had the heterozygous CNV. The genetic causes of Family P31 and P34 were exon 3 del and exon 3–5 del in *OPN1LW*, respectively, these two CNVs were novel.

## Discussion

This study selected a cohort of 35 unsolved IRD cases by NGS. Further, MLPA, QF-PCR, Sanger sequencing were employed to screen CNVs in these cases. In total, 33 different CNVs in 16 IRD-associated genes were found (Table S2), 26 and 7 of them were deletions and duplication CNVs, respectively. Herein, *PRPF31* (five different CNVs in six cases), *EYS* (five different CNVs in four cases), *USH2A* (4 different CNVs in four cases) were the most common disease-causing gene in CNVs. And 14 novel variants in 10 different IRD genes were detected, enriching IRDs mutational spectrums, emphasizing the fact that CNVs contributed a lot to genetic causality of IRDs.

Our study reminds of us that CNV surveillance should be applied into first-tier gene panel NGS services (Bujakowska et al. 2017; Ellingford et al. 2018), especially for those CNV-prone IRD genes, such as *PRPF31*, *EYS*, *USH2A*, etc. Previously, a study (Van Schil et al. 2018) conducted an extensive literature mining of reported CNVs in all known IRD genes listed in RetNet and retrieved 1,345 CNVs in 81 different genes from 300 different publications to prioritize those genes prone to CNVs. They listed the 15 top-ranked genes according to the total CNV count, together with the number of unique CNVs in the corresponding gene, including *USH2A*,* RS1*, *OPN1LW*/*OPN1MW*, *EYS*, ABCA4 and PRPF31, and so on, consisting with the results reported herein and by our previous studies (Liu et al. 2021). They also demonstrated the strongest correlation between gene size and CNV occurrence of IRD genes. Herein, the top two common with CNVs in Chinese IRDs patients were *EYS* and *USH2A*, which are two of the largest IRD genes with a high prevalence in the Asian and global population (Gao et al. 2021; Pontikos et al. 2020; Su et al. 2022; Yang et al. 2020). We wonder whether high prevalence is also the reason why CNVs are prone to occurred in these genes. Presently, haploinsufficiency has been suggested to be the main pathological mechanism of *PRPF31*‐associated adRP (Abu-Safieh et al. 2006; Vithana et al. 2003; Wheway et al. 2020), and *PRPF31* frequently harbors CNVs (Chen et al. 2022; Zampaglione et al. 2020). *PRPF31* is located on a region rich in repeat elements, especially in Alu repeats. Alu elements are a type of short interspersed element that accounts for 10% of the total genome content, but in chromosome 19 accounts for 26.3%, with Alu being the richest chromosome (Grover et al. 2004). A previous study (Chen et al. 2022) also provided strong evidence that Alu elements of *PRPF31* probably contributed to the susceptibility to genomic rearrangement in this locus. Herein, we reported several CNVs (exon 1–14 del, exon2-14 del, exon 2–3 del and exon 14 exon del) in *PRPF31*, consisting with the above points. In summary, the occurrence of CNVs could be expected in genes with large gene size and complex repeats, although CNVs in some of them have not been detected yet, an important reason is that CNV screening has not been routinely implemented, likely leading to an underestimation of the CNV mutational load in these genes (Van Schil et al. 2018).

In total, we confirmed 33 CNV different events, which consist of 26 deletion CNVs and 7 duplication CNVs in 16 different genes through the described methods, including 1 same whole gene deletion CNV in 3 families. These data suggest that IRD genes are more susceptible to deletion than duplication, consistent with the results reported by Ellingford et al. (2018). While Ellingford et al*.* declare their observations may be a limitation of the approaches applied, as NGS read-depth CNV detection software is less sensitive for small duplication events (Ellingford et al. 2018; Fowler et al. 2016), and duplications also prove more challenging for clinical interpretation as we were unable to determine phase of apparently homozygous events or confirm the genomic location of duplicated sequences. Previously, we have reported our tool and an additional sample filtering programme to improve CNV detection ability, in which the samples with low data quality would be removed (Liu et al. 2021). The detection of CNVs from high-coverage NGS data provides a unique opportunity for the simultaneous analysis of novel disease-causing SNVs and small indels (Ellingford et al. 2016). Many informatics techniques exist to identify CNVs from NGS data sets. At the same time, gene panel NGS approaches are limited by the types of CNV detection algorithms that can be routinely applied (Pirooznia et al. 2015). HEDEP achieved a higher sequencing depth (up to 800×) than WES, increasing the CNV detection rate theoretically and practically. As shown in Fig. S1, duplication CNV in the *NDP* gene and deletion CNVs in *GPR143* and *ABCA4* genes were detected by HEDPE during bioinformatic analysis. However, HEDEP may face the same challenge to detect duplication CNVs with more difficulty.

Currently, various techniques are available for identifying genomic CNVs, including MLPA, QF-PCR, genome-wide and customized array CGH, and low-coverage genome-wide sequencing (Alkan et al. 2011), even Sanger sequencing. When filtering CNVs, we should consider the issues, including turnaround time, financial cost, human cost, disease features, etc. Initially, we used MLPA to validate CNVs detected by HEDEP. MLPA is used as a first-line test to detect CNV of specific genes and has many merits (Stuppia et al. 2012): (a) MLPA is a high throughput analysis, allowing up to 96 samples to be handled simultaneously, with results being available within 24 h; (b) MLPA is a multiplex technique, allowing the study of several regions of the human genome in a single reaction; (c) The MLPA reaction can also be carried out on DNA extracted from a buccal swab, providing an easier system of sample collection compared to peripheral blood withdrawal; (d) MLPA is a low cost and technically uncomplicated method compared to array CGH. So far, MLPA kits are commercially available from MRC Holland (https://www.mrcholland.com). While for CNV filtering, the financial and human cost of MLPA is higher than that of QF-PCR. In this study, five cases with red-green CVD family history were subjected to HEDEP, no disease-causing SNVs/indels in IRD genes were obtained. Variants in the *OPN1LW*/*OPN1MW* gene cluster cause a range of mild to severe X-linked CVD, including red-green CVD or deuteranopia with normal visual acuity and cone ERG, affects approximately 1 in 12 males and 1 in 200 females(Mizrahi-Meissonnier et al. 2010; Neitz et al. 1996). For patients with a definite medical history or family history of CVD, Sanger sequencing, which is cost-effective and time-saving rather than MLPA, can be employed to further screen CNVs in these two genes. To have healthy children, two unaffected female individuals (P33, individual IV:1 and P35, II:1) were willing to employ genetic testing to screen pathogenic variants because of their affected family numbers. This indicates the gradually increasing acceptance of genetic diagnosis and emphasizes the importance of CNV filtrating.

Herein, several probands with a heterozygous SNV or small indel in AR IRD genes, deep-intronic variants, and CNVs should be analyzed. For patients with homozygous disease-causing variants in AR IRD genes, it is essential to confirm the patient’s family history and inheritance pattern, if their parents were not consanguineous, deletion CNVs in another allele should be considered. A previous study (Ellingford et al. 2018) reported a heterozygous whole gene deletion of *RPE65* was identified for an individual originally described with a pathogenic homozygous missense variant (c.1102 T > C; p.Tyr368His), and subsequent familial segregation analysis confirmed these events to be paternally and maternally inherited, respectively. This indicates confirmation of *in-trans* variants included the encapsulation of a homozygous SNV/indel by a heterozygous deletion event or familial segregation analysis. Similarly, CNV in *CYP4V2* (exon 1–11 del), *PCARE* (exon 1–2 del), *RS1* (exon 4–6 del) to make the disease-causing missense variant (c.802-8_810del17bpinsGC in IVS6 of *CYP4V2*, c.644delT in exon 1 of *PCARE*, c.214G > T in exon 4 of *RS1*) in these genes homozygous, respectively. It is worth noting that the proband of Family P26 was a 6-year-old girl who suffered from macular retinoschisis, the WES result showed she had the homozygous variant c.214G > T (p.Glu72*) in *RS1,* which is located on the X chromosome. According to ACMG guideline, this variant was pathogenic, while her parents were healthy and did not have pathogenic variants. Different from male, female has two *RS1* allele, she acquired the same variant from parents with a slight chance becasue her parents were not consanguinous. This result did not elucidate the genetic cause of her macular retinoschisis phenotype. Further, QF-PCR indicated that she had a heterozygous CNV in *RS1*, which was not inherited from her parents either. These results showed that her phenotypes were caused by the compound variants in *RS1*. However, we wonder whether she caught these two variants spontaneously, or although her parents did not carry the variant in peripheral blood DNA, one of the parents must be a mosaic for that variant of their germ cells. Besides, our study also emphasizes the significance of co-segregation analysis in available family numbers when filtering CNVs. Take Family P02 as an example, two CNVs in the proband were detected by HEDEP and MLPA, however, the methods could not confirm they were located on one allele or two alleles. If these two CNVs were *in cis* on one allele, they could not lead to her phenotypes. Co-segregation analysis showed that one of the CNVs—exon 13–15 del in *EYS *were passed to her daughter and son, showing they were compound heterozygous and were disease-causing.

In summary, we demonstrate that CNVs significantly contribute towards the occurrence of IRD and emphasize that CNV surveillance should be applied to first-tier gene panel NGS services. The detection of novel CNVs expands the IRD mutational spectrum.

### Supplementary Information

Below is the link to the electronic supplementary material.

Supplementary file1 (PDF 701 KB)

## Data Availability

The data that support the findings of this study are available from the corresponding author on reasonable request.
