# Supplementary material for: Screening copy number variations in 35 unsolved inherited retinal disease families
Source: Hum Genet. 2024 Jan 29;143(2):197–210. doi: 10.1007/s00439-023-02631-4 (PMC10881639; doi:10.1007/s00439-023-02631-4)
Supplement: Supplementary file 1 — Supplementary file1 (PDF 701 KB) [file 439_2023_2631_MOESM1_ESM.pdf]

**Supplemental Data**

# Screening Copy Number Variations in 35 unsolved Inherited Retinal Disease Families

Xiaozhen Liu, Hehua Dai, Genlin Li, Ruixuan Jia, Xiang Meng, Shicheng, Yu, Liping Yang, Jing Hong

Xiaozhen Liu and Hehua Dai contribute to this article equally.

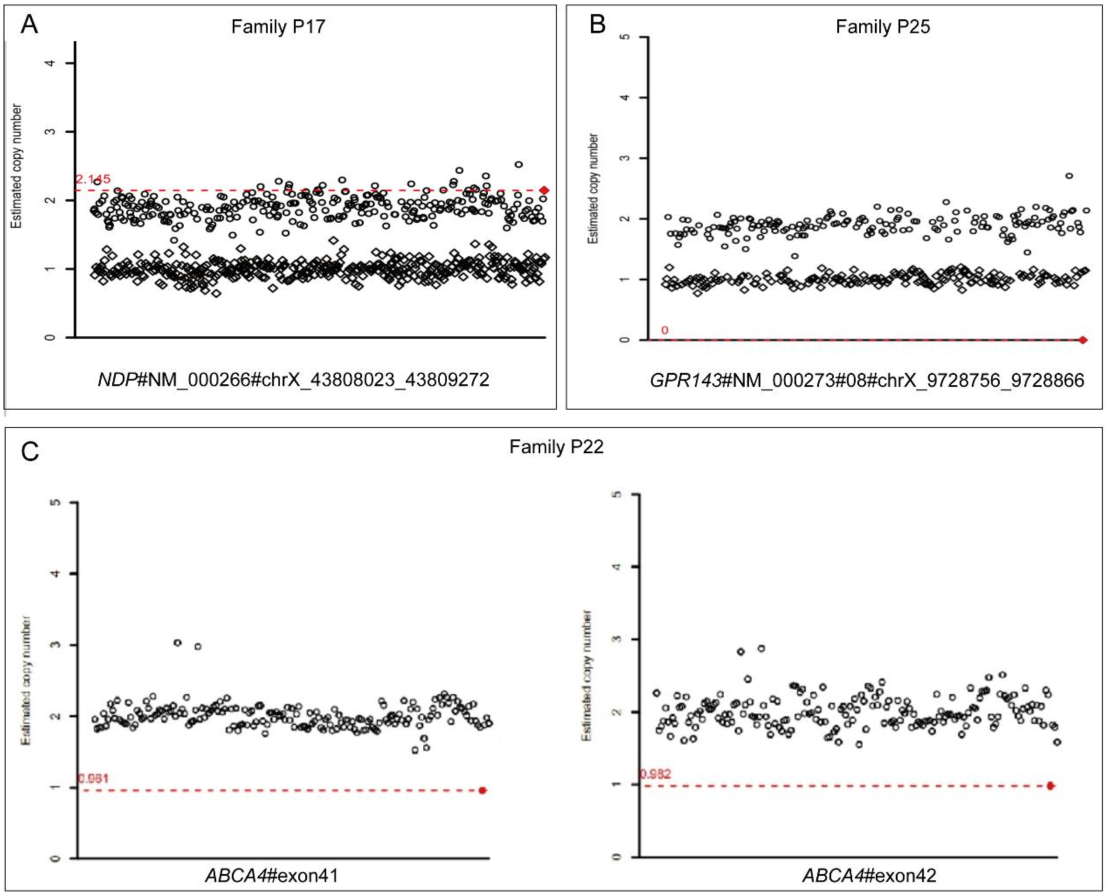


**Fig. S1-revised. Copy number variations predicted by HEDEP.**

Arrange from left to right on the X-axis by next generation sequencing time.

The Y-axis is the number of copies of IRD genes estimated by the algorithm. Each black point in the figure represents a healthy control sample subjected to HEDEP. Red dotted line represents the patient sample subjected to HEDEP. HEDEP results indicated the proband of (A) Family P17 had a hemizygous duplication CNV (exon 3 dup; #NM_000266#chrX_43808023_43809272) in *NDP*; (B) Family P25 had a hemizygous CNV (exon 3 del; #NM_000273#chrX_9728756_9728866) in *GPR143*; (C) Family P25 had a heterozygous CNV (exon 41-41 del; #NM_000350.3#exon 41 and 42) in *ABCA4.*

**Table S1**. Primer's list

| | Forward primer (5'-3') | Reverse primer (5'-3') | Length |
|---|---|---|---|
| **quantitative PCR** | | | |
| ALB | GGTGTTGATTGCCTTTGCTC | CAGCTGACTCATCAGCAACACAT | 113bp |
| ABCA4_E41 | GATTGCCGAGCCCACTAAGGA | GGTTAGTTCATGTAGCCTTAAG | 118bp |
| ABCA4_E42 | ATTTATCCAGGCACCTCCAGC | CTCTCCAGGGCGAACTC | 63bp |
| ADGRV1_86 | ATTCCAAACGTCTATGCTGCTT | CTGCAGCATTTGTCCTTCCTCT | 151bp |
| PCARE_E1 | TGCAGGGTCTCCCCTTATGCT | GAAGTCACCGACTTCCATTCTTCG | 136bp |
| PCARE_E2 | ACCCAGAGATGATGGGCAAACC | TTTGAACCCCAAGGCAGAGCAAG | 138bp |
| CRB1_E1 | CTGTGAAGGAGCTGTAAGTAGG | GAGAACATCCTCTGGTGTGTTG | 136bp |
| CRB1_E3 | GACGAATGTTGGTCCCAGCCTTG | CATGGAGACAAGGTTGACTGGCAC | 145bp |
| GPR143_E2 | TTATCCAATTCTCTCTTAGGTATGGTGATC | TTGCTGCTGCTGCGATTTGAGGA | 195bp |
| PROM1_E15 | TGACTGCAAAAAAAATAGAGGCAC | CTCATTAATGTTGAGATGTTCAC | 85bp |
| PROM1_E16 | GGAAGCATAAGCAGTGAATTG | CTGAGCCAAGTAGCTGTCAT | 138bp |
| PROM1_E17 | ACTGGTAAATCCCCCGCAG | CAAACTGTTTGCTTTTGCTTC | 72bp |
| PROM1_E18 | CCAGGAAATTTGAGGAACTCC | CAGTGATTGTTCTATAGGAAG | 90bp |
| PROM1_E19 | AGCACTCTATACCAAAG | CAACAATCCATTCCCTGTG | 54bp |
| PROM1_E20 | GAGAGAGTAACTAGGATTCTAG | CTCAATAATAACAGAGG | 81bp |
| PROM1_E21 | GAAACTAAGAAGTATGGGA | AGAGAACTCGATCCACTGCA | 69bp |
| PROM1_E22 | ATCAGTGAGAAAGTGGCATCG | CAAGGGGTCGATAATGTAGCT | 93bp |
| PROM1_E23 | GGTTTGGCATAGGAAAAGCTA | CATCGTACACGTCCTCCGA | 105bp |
| PROM1_E24 | TGTTGAAACTATACCCATGA | GTTAATAACTTAATTTTTAAAG | 113bp |
| PROM1_E25 | TATGGAAAATGGTAATAATG | CTTGTCATAACAGGATTGTGAA | 69bp |
| RLBP1_E3 | TTTCTAACCCCTGCAGCCCTGA | CTTACCCCTTCTGACATGTTGCC | 133bp |
| RLBP1_E4 | CGCATGGTACCTGAAGAGGAACA | TCAGCCACCTCACCTTCTGCAA | 130bp |
| RS1_E1 | GCCAAAGACCTAAGAACTAAATGG | AAAGCCTTCTATCTTGCGTGAC | 148bp |
| RS1_E2 | CCACATTGGGATTATCGTCT | CCCAGCCAAAATATATTTCA | 150bp |
| RS1_E3 | GATGAAGGCGAGGACCCCTGGT | CCTCCTTGGACTGTATACCAG | 106bp |
| RS1_E4 | GAATGCCCATATCACAAGCCTC | CTGCTTACCCAAAGCCTTGAC | 151bp |
| RS1_E5 | GTGCCTGGCTCTCCAAGTT | CTGGTCCTTGTAGTAAATCCAG | 179bp |
| RS1_E6 | GTCTTCTATGGCAACTCGGAC | GCAGGCATCAGGCACACTTG | 160bp |
| NDP_E3 | GCCTTTGGTGTCGTTCAGCACT | GTAGGTGGCAGTGAGTCGCAT | 133bp |
| NYX_E1 | AAGAAGTGTGGAGGCATGGG | CTCACCATGCAGAAGCAGGA | 158bp |
| USH2A_E22 | GTATGCATGCTTGTATCAGGATC | TTGTAGTTACTTCCACTGGTGAC | 147bp |
| USH2A_E23 | TGATTGTCTTTGCAGCATCACCTG | TTTACCTCAGTACCAGGCACCTA | 157bp |
| USH2A_E25 | GGCAGAGTTCTGAAGAACAAATC | GGTCAAGTTAATCAAACAGGAGAG | 180bp |
| USH2A_E23 | GGGTCACCAGTGGAAGTAAC | AACACCAACATATCAACAGGGC | 176bp |
| USH2A_E24 | CTCTAGGTTCCTCTGCCATCCT | GATGGTATAACTTCGCGGGAGC | 92bp |
| **PCR and Sanger Sequencing** | | | |
| OPN1LW/MW_E1 | CCCAGGCCCAATTAAGAGAT | GGGACGTGCAGAAGAGAGAT | 387bp |
| OPN1LW_E2 | TGGACAAAGCTGGAGGGAAA | ATATGGATGTGAGGCGCAGAT | 568bp |
| OPN1MW_E3 | TGCAGACGTTTGGGGTCTAA | TCCTATGTTGCAGCCACATT | 394bp |
| OPN1LW_E4 | GCCACAGAATTGATCACTTCA | AGTGGACTCATTTGAGGGCA | 398bp |

| | | | |
|---|---|---|---|
| OPN1MW_E5 | TCCACTCAGGGCTGGAAGAT | CGGGCTTCTTATCAGAGACAT | 460bp |
| OPN1LW_E6 | TTCAACCCAGTGTAGTCACCA | TTTTACAGGGATGGAGAAGGA | 377bp |

**Table S2.** 35 Chinese IRDs families with (likely) causative copy number variants

| Family ID | Clinical diagnosis | Proband's gender | Age(year) | | Type | Variant(s) detected by NGS | | | | | | | | | CNVs validated by an additional method | | | | | | |
|---|---|---|---|---|---|---|---|---|---|---|---|---|---|---|---|---|---|---|---|---|---|
| | | | Onset | Examination | | Testing method | Gene | Transcript ID | Genotype | exon/intron | Sequence Change | | Literature | ACMG classificatio | Testing method | Gene | Transcript ID | Genotype | CNVs | Literature | ACMG classificatio |
| P01 | CORD | F | 8 | 54 | Sporadic | HEDEP | *USH2A* | NM_206933.3 | Het | exon68 | c.14876G>A | p.Gly4959Asp | Liu et al. (2020) | P | MLPA | *USH2A* | NM_206933.3 | Het | exon 11-21 del | Liu et al. (2021) | P |
| P02 | CORD | F | 10 | 42 | Sporadic | HEDEP | - | - | - | - | - | - | - | - | MLPA | *EYS* | NM_001142800.2 | Com.Het | exon 6-7 del | Liu et al. (2021) | P |
| | | | | | | | | | | | | | | | | | | | exon 13-15 del | Liu et al. (2021) | P |
| P03 | RP | F | 5 | 45 | Sporadic | HEDEP | *EYS* | NM_001142800.2 | Het | exon6 | c.919G>T | p.Gly307* | Liu et al. (2020) | P | MLPA | *EYS* | NM_001142800.2 | Het | exon 31-32 del | Liu et al. (2021) | P |
| P04 | RP | M | Childhood | 16 | Sporadic | HEDEP | - | | - | - | - | - | - | - | MLPA | *PRPF31* | NM_015629 | Het | exon 2-14 del | Liu et al. (2021) | P |
| P05 | STGD | F | Childhood | 11 | Sporadic | HEDEP | *ABCA4* | NM_000350.3 | Het | exon19 | c.2909C>T | p. Thr970Ile | Stenirri et al. (2008) | P | MLPA | *ABCA4* | NM_000350.3 | Het | exon 38-44 dup | Liu et al. (2021) | P |
| P06 | Choroideremia | M | 12 | 49 | Sporadic | HEDEP | - | | - | - | - | - | - | - | MLPA | *PRPF31* | NM_015629 | Het | exon 1-14del | Liu et al. (2021) | P |
| P07 | RP | M | 10 | 44 | AD | HEDEP | - | | - | - | - | - | - | - | MLPA | *PRPF31* | NM_015629 | Het | exon 2-3 del | Liu et al. (2021) | P |
| P08 | RP | M | 6 | 18 | Sporadic | HEDEP | - | | - | - | - | - | - | - | MLPA | *PRPF31* | NM_015629 | Het | exon 2-13 del | Liu et al. (2021) | P |
| P09 | CORD | F | 7 | 24 | Sporadic | HEDEP | *EYS* | NM_001142800.2 | Het | exon26 | c.4655T>A | p.Leu1552* | Liu et al. (2020) | P | MLPA | *EYS* | NM_001142800.2 | Het | exon 15 del | Liu et al. (2021) | P |
| P10 | USH | F | 15 | 54 | Sporadic | HEDEP | - | | - | - | - | - | - | - | MLPA | *PRPF31* | NM_015629 | Het | exon 14 del | Liu et al. (2021) | P |
| P11 | BCD | M | 10 | 41 | AR | HEDEP | *CYP4V2* | NM_207352 | Homo | IVS6 | c.802-8_810del17bpinsGC | - | Xiao et al. (2011) | P | MLPA | *CYP4V2* | NM_207352 | Het | exon 1-11 del | Liu et al. (2021) | P |
| P12 | RP | M | 4 | 26 | Sporadic | HEDEP | *CRB1* | NM_201253.3 | Het | exon7 | c.2234C>T | p.Thr745Met | den Hollander et al. (1999) | P | MLPA | *CRB1* | NM_201253.3 | Het | exon 6-8 dup | Liu et al. (2021) | P |
| P13 | RP | M | 14 | 60 | Sporadic | HEDEP | - | | - | - | - | - | - | - | MLPA | *PRPF31* | NM_015629 | Het | exon 14 del | Liu et al. (2021) | P |
| P14 | CORD | M | 10 | 52 | AD | HEDEP | *EYS* | NM_001142800.2 | Het | exon42 | c.8143C>T | p.Arg2715* | Liu et al. (2020) | P | MLPA | *EYS* | NM_001142800.2 | Het | exon 14-22 del | Liu et al. (2021) | P |
| P15 | USH | F | 4 | 18 | Sporadic | HEDEP | *USH2A* | NM_206933.3 | Het | exon61 | c.11762_11787 del26bp | p.Ala3922His fs*116 | This study | LP | MLPA | *USH2A* | NM_206933.3 | Het | exon 47 del | Le Quesne Stabej et al.(2012) | P |
| P16 | RP | M | 20 | 34 | AR | HEDEP | *PROM1* | NM_001145848 | Het | exon1 | c.139delC | p.His47Ilefs*12 | Xu et al. (2014) | P | qPCR | *PROM1* | NM_001145848 | Het | exon 15-25 dup | Liu et al. (2021) | P |
| P17 | FEVR | M | 7 | 29 | XL | HEDEP | - | | - | - | - | - | - | - | qPCR | *NDP* | NM_000266 | Hemi | exon 3 dup | Liu et al. (2021) | P |
| P18 | Retinoschisis | M | 1 | 4 | Sporadic | HEDEP | - | | - | - | - | - | - | - | qPCR | *RS1* | NM_000330.4 | Hemi | exon 1-3 del | Liu et al. (2021) | P |
| P19 | USH | F | 14 | 39 | Sporadic | HEDEP | *USH2A* | NM_206933.3 | Het | exon 53 | c.10514C>G | p.Pro3505Arg | This study | US | MLPA | *USH2A* | NM_206933.3 | Het | exon 61 dup | This study | P |
| P20 | RP | M | 10 | 50 | Sporadic | HEDEP | *PCARE* | NM_001029883.3 | Homo | exon1 | c.644delT | p.Leu215Argfs*41 | This study | LP | qPCR | *PCARE* | NM_001029883.3 | Het | exon 1-2 del | This study | P |
| P21 | RP | M | 22 | 48 | AR | HEDEP | *ADGRV1* | NM_032119.4 | Het | exon79 | c.17187C>A | p.Cys5729* | Jiao Q et al. (2019) | LP | qPCR | *ADGRV1* | NM_032119.4 | Het | exon 86 dup | This study | P |
| P22 | STGD | F | 11 | 15 | Sporadic | HEDEP | *ABCA4* | NM_000350.3 | Het | exon36 | c.5163dupC | p.Thr1722Hisfs*65 | This study | LP | qPCR | *ABCA4* | NM_000350.3 | Het | exon 41-42 del | This study | P |
| P23 | RP | M | 4 | 7 | Sporadic | HEDEP | *CRB1* | NM_201253.3 | Het | exon7 | c.2291G>A | p.Arg764His | Corton M et al. (2013) | LP | qPCR | *CRB1* | NM_201253.3 | Het | exon 1 del | This study | P |
| P24 | Macular Retinoschisis | M | 4 | 4 | Sporadic | HEDEP | *CRB1* | NM_201253.3 | Het | IVS5 | c.1171+1G>A | - | This study | LP | qPCR | *CRB1* | NM_201253.3 | Het | exon 3 del | This study | P |
| P25 | CN? | M | 1 | 31 | Sporadic | HEDEP | - | | - | - | - | - | - | - | qPCR | *GPR143* | NM_000273 | Hemi | exon 2 del | This study | P |
| P26 | Macular Retinoschisis | F | 4 | 6 | Sporadic | WES | *RS1* | NM_000330.4 | Homo | exon4 | c.214G>T | p.Glu72* | This study | P | qPCR | *RS1* | NM_000330.4 | Het | exon 4-6 del | This study | P |
| P27 | Macular Retinoschisis | M | 27 | 27 | Sporadic | HEDEP | - | | - | - | - | - | - | - | qPCR | *RS1* | NM_000330.4 | Hemi | exon 1 del | This study | P |
| P28 | RP | M | 15 | 50 | Sporadic | HEDEP | *RLBP1* | NM_000326 | Het | IVS7 | c.684+1G>C | - | This study | LP | qPCR | *RLBP1* | NM_000326 | Het | exon 3-4 dup | This study | P |
| P29 | RP | F | childhood | 48 | AR | HEDEP | - | | - | - | - | - | - | - | qPCR | *USH2A* | NM_206933.3 | Homo | exon 22-24 del | This study | P |
| P30 | RP | M | 1 | 2 | AD | WES | *ABCA4* | NM_000350.3 | Com. Het | exon42 | c.5881G>A | p.Gly1961Arg | Riveiro-Alvarez R et al.(2013) | LP | qPCR | *NYX* | NM_022567 | Hemi | exon 1 del | This study | P |
| | | | | | | | | | | exon11 | c.1405G>C | p.Gly469Arg | This study | US | | | | | | | |
| P31 | CVD | M | childhood | 41 | XL | HEDEP | - | | - | - | - | - | - | - | Sanger | *OPN1LW* | NM_020061 | Hemi | exon 3 del | This study | P |
| P32 | CVD | M | 5 | 5 | XL | HEDEP | - | | - | - | - | - | - | - | Sanger | *OPN1MW* | NM_000513.2 | Hemi | entire gene del | Ayyagari R et al.(2000) | P |
| P33 | CVD | M | childhood | 58 | XL | HEDEP | - | | - | - | - | - | - | - | Sanger | *OPN1MW* | NM_000513.2 | Hemi | entire gene del | Ayyagari R et al.(2000) | P |
| P34 | CVD;Deafness | M | childhood | 32 | Sporadic | HEDEP | *GJB2* | NM_004004.6 | Homo | | c.235delC | p.Leu79Cysfs*3 | Xia H et al. (2019) | P | Sanger/qF | *OPN1LW* | NM_020061 | Hemi | exon 3-5 del | This study | P |
| P35 | CVD | M | childhood | 57 | Sporadic | HEDEP | - | | - | - | - | - | - | - | Sanger | *OPN1MW* | NM_000513.2 | Het | entire gene del | Ayyagari R et al.(2000) | P |

AD= autosomal dominant; AR= autosomal recessive; BCD= Bietti Crystalline Corneoretinal Dystrophy; CORD= Cone-rod Dystrophy; CN= Congenital nystagmus; CVD= Color Vision Deficiency; F=Female; FEVR= Familial Exudative Vitreoretinopathy; HEDEP= Hereditary Eye Disease Enrichment Panel; Het= heterozygous; Hemi= Hemizygous; Homo= homozygous; Com. Het= Compoumd heterozygous; LP= P= likely pathogenic; LCA=Leber Congenital Amaurosis; M=Male; MD=Macular Degeneration; MLPA= Multiplex Ligation-Dependent Probe Amplification; NGS= Next generation sequencing; P= pathogenic; qPCR= quantitative Polymerase Chain Reaction; RP= Retinitis Pigmentosa; STGD=Stargardt Disease; XL= X-Linked; US = uncertain significance; WES= the Whole Exome Sequencing

**Table S3.** The classification of CNVs according to ACMG and the AMP variant interpretation guidelines

| Family ID | CNVs validated by an additional method | | | Evidence of pathogenicity | | | | | | | | | | | | | | | |
|---|---|---|---|---|---|---|---|---|---|---|---|---|---|---|---|---|---|---|---|
| | Gene | CNVs | ACMG classification | Very strong | Strong | | | | Moderate | | | | | | Supporting | | | | |
| | | | | PVS1 | PS1 | PS2 | PS3 | PS4 | PM1 | PM2 | PM3 | PM4 | PM5 | PM6 | PP1 | PP2 | PP3 | PP4 | PP5 |
| P01 | *USH2A* | exon 11-21 del | P | Y | Y | | | | | | | Y | | | Y | | Y | | |
| P02 | *EYS* | exon 6-7 del | P | Y | Y | | | | | | | Y | | | | | Y | | |
| | | exon 13-15 del | P | Y | Y | | | | | | | Y | | | | | Y | | |
| P03 | *EYS* | exon 31-32 del | P | Y | Y | | | | | | | Y | | | | | Y | | |
| P04 | *PRPF31* | exon 2-14 del | P | | Y | Y | | | | | | Y | | | | | Y | | |
| P05 | *ABCA4* | exon 38-44 dup | P | Y | Y | | | | | | | | | | Y | | Y | | |
| P06 | *PRPF31* | exon 1-14del | P | | Y | | | | Y | | | Y | | | | | Y | | |
| P07 | *PRPF31* | exon 2-3 del | P | | Y | | | | Y | | | Y | | | Y | | Y | | |
| P08 | *PRPF31* | exon 2-13 del | P | | Y | | | | | | | Y | | | | | Y | | |
| P09 | *EYS* | exon 15 del | P | Y | Y | | | | | | | Y | | | Y | | Y | | |
| P10 | *PRPF31* | exon 14 del | P | | Y | | | | Y | | | Y | | | | | Y | | |
| P11 | *CYP4V2* | exon 1-11 del | P | Y | Y | | | | | | | Y | | | Y | | Y | | |
| P12 | *CRB1* | exon 6-8 dup | P | Y | Y | | | | | | | | | | Y | | Y | | |
| P13 | *PRPF31* | exon 14 del | P | | Y | | | | Y | | | Y | | | | | Y | | |
| P14 | *EYS* | exon 14-22 del | P | Y | Y | | | | | | | Y | | | | | Y | | |
| P15 | *USH2A* | exon 47 del | P | Y | Y | | | | | | | Y | | | Y | | Y | | |
| P16 | *PROM1* | exon 15-25 dup | P | Y | Y | | | | | | | | | | Y | | Y | | |
| P17 | *NDP* | exon 3 dup | P | Y | Y | | | | | | | | | | | | Y | | |
| P18 | *RS1* | exon 1-3 del | P | Y | Y | | | | Y | | | Y | | | | | Y | | |
| P19 | *USH2A* | exon 61 dup | P | Y | | | | | | Y | | | | | Y | | Y | | |
| P20 | *PCARE* | exon 1-2 del | P | Y | | | | | | Y | | Y | | Y | Y | | Y | | |
| P21 | *ADGRV1* | exon 86 dup | P | Y | | | | | | Y | | | | | Y | | Y | | |
| P22 | *ABCA4* | exon 41-42 del | P | Y | | | | | | Y | | Y | | Y | | | Y | | |
| P23 | *CRB1* | exon 1 del | P | Y | | | | | | Y | | Y | | | Y | | Y | | |
| P24 | *CRB1* | exon 3 del | P | Y | | | | | | Y | | Y | | | Y | | Y | | |
| P25 | *GPR143* | exon 2 del | P | Y | | | | | | Y | | Y | | | Y | | Y | | |
| P26 | *RS1* | exon 4-6 del | P | Y | | Y | | | | Y | | Y | | | | | Y | | |
| P27 | *RS1* | exon 1 del | P | Y | | | | | | Y | | Y | | | | | Y | | |
| P28 | *RLBP1* | exon 3-4 dup | P | Y | | | | | | Y | | | | | Y | | Y | | |
| P29 | *USH2A* | exon 22-24 del | P | Y | | | | | | Y | | Y | | | Y | | Y | | |
| P30 | *NYX* | exon 1 del | P | Y | | | | | | Y | | Y | | | Y | | Y | | |
| P31 | *OPN1LW* | exon 3 del | P | Y | | | | | | Y | | Y | | | | | Y | | |
| P32 | *OPN1MW* | entire gene del | P | Y | Y | | | | | | | Y | | | | | Y | | |
| P33 | *OPN1MW* | entire gene del | P | Y | Y | | | | | | | Y | | | Y | | Y | | |
| P34 | *OPN1LW* | exon 3-5 del | P | Y | | | | | | Y | | Y | | | | | Y | | |
| P35 | *OPN1MW* | entire gene del | P | Y | Y | | | | | | | Y | | | Y | | Y | | |

Y indicates the affected family has the evidence of pathogenicity
